# Back Numbers

**Published:** 1868-03-01

**Authors:** 


### EDITORIAL.

---

### *Back Numbers.*

Thanks to the kind responses of our friends, we have received a sufficient number of Journals to complete the files of all who have applied for missing numbers in 1867. No more copies are needed, and if any who have written for extra ones have not received them, by dropping us a line they will be forwarded by return mail.

